# Efficient embryoid-based method to improve generation of optic vesicles from human induced pluripotent stem cells

**DOI:** 10.12688/f1000research.108829.1

**Published:** 2022-03-17

**Authors:** Jonathan Eintracht, Philippa Harding, Dulce Lima Cunha, Mariya Moosajee

**Affiliations:** 1 Institute of Ophthalmology, University College London, London, EC1V 9EL, UK; 2 The Francis Crick Institute, London, NW1 1AT, UK; 3 Moorfields Eye Hospital NHS Foundation Trust, London, EC1V 2PD, UK; 4 Great Ormond Street Hospital for Children, London, WC1N 3JH, UK

**Keywords:** Embryoid bodies, optic vesicles, iPSCs, retinal differentiation, VSX2, PAX6, eye development

## Abstract

Animal models have provided many insights into ocular development and disease, but they remain suboptimal for understanding human oculogenesis. Eye development requires spatiotemporal gene expression patterns and disease phenotypes can differ significantly between humans and animal models, with patient-associated mutations causing embryonic lethality reported in some animal models. The emergence of human induced pluripotent stem cell (hiPSC) technology has provided a new resource for dissecting the complex nature of early eye morphogenesis through the generation of three-dimensional (3D) cellular models. By using patient-specific hiPSCs to generate *in vitro *optic vesicle-like models, we can enhance the understanding of early developmental eye disorders and provide a pre-clinical platform for disease modelling and therapeutics testing. A major challenge of *in vitro *optic vesicle generation is the low efficiency of differentiation in 3D cultures. To address this, we adapted a previously published protocol of retinal organoid differentiation to improve embryoid body formation using a microwell plate. Established morphology, upregulated transcript levels of known early eye-field transcription factors and protein expression of standard retinal progenitor markers confirmed the optic vesicle/presumptive optic cup identity of *in vitro *models between day 20 and 50 of culture. This adapted protocol is relevant to researchers seeking a physiologically relevant model of early human ocular development and disease with a view to replacing animal models.

Research highlights **Scientific benefit(s)**

• Generation of a highly faithful model of human early ocular development
• More physiologically and pathologically relevant model than animals
• Ability to generate patient-specific models of disease with a view to personalised medicine

**3Rs benefits(s)**

• Replacement of animal models of early ocular development and disease with human induced pluripotent stem cell-derived models

**Practical benefits(s)**

• Circumvent ethical considerations associated with embryonic stem cells
• The generation, expansion and storage of hiPSCs is relatively straightforward
• Easier to access than human foetal tissue

**Current applications**

• Development and disease modelling, therapeutics testing

**Potential applications**

• Platforms for pre-clinical gene, cell and small molecule therapy development
• High-throughput drug screening systems
• Use in creating complex models of the eye, such as organ-on-a-chip technologies

## Introduction

Eye development is a well-studied, highly-conserved process across humans and animal models. ^1^ Consequently, many of the molecular mechanisms and genetic networks underlying eye morphogenesis have been clearly described in the literature. ^2^^,^^3^ In early oculogenesis, eye-field transcription factors (EFTFs) involved in the eye specification, including *RAX* and *PAX6* are essential for optic vesicle formation at ~3-4 weeks of embryonic development and subsequent invagination to form the optic cup at ~5 weeks. Later, the visual homeobox system 2 gene ( *VSX2*) is required for establishing retinal progenitor cells in the presumptive optic cup. The role and regulation of these genes discovered in animal models have greatly informed our understanding of human ocular development, and how dysregulation of these factors can lead to developmental eye disorders. ^2^

From 2019-2021, approximately 12000 peer-reviewed journal articles investigating ocular development and disease were published, with roughly 2000 articles investigating ocular development and disease using animal models including zebrafish, *Xenopus*, mice, rats, dogs, cats and chicks. Approximately half of these papers investigated early ocular development using animals that could be replaced with a human stem-cell based model. The number of animals used varies greatly between studies due to a variety of experimental variables, however, the average is around 50 throughout the literature. This equates to roughly 100,000 animals used for experimental procedures, without considering those used for optimisation. To generate a CRISPR/Cas9-induced mutation, at least 750 adult zebrafish are required to test the efficiencies of the single guide RNAs (sgRNAs) and generate a mixed population of homozygous, heterozygous and wild type fish that can be genotyped. Homozygous fish can be phenotyped as an exclusive population with the introduced mutation. Additionally, any attempt to knockdown a gene in zebrafish using morpholinos requires at least 200 embryos per candidate gene. For context, in our laboratory, a total of 3040 zebrafish were used in the past year. Most experiments on typical animal models such as rats, zebrafish or chicks would be classified as non-recovery such as enucleation of the eye for histology, immunohistochemistry or RNA extraction, or as severe due to general anaesthesia and surgical techniques such as sub-retinal cell transplantation performed on the animals. There are many groups investigating ocular development and disease using animal models that would benefit from adopting a stem cell-based technique which does not compromise the scientific integrity of their disease models, in line with the principles of the 3Rs; replacement, refinement and reduction of animals in research.

Despite their invaluable contribution, animal models are suboptimal for studying human eye development and disease for several critical reasons: (i) Ocular morphogenesis differs between humans and animal models. ^4^ (ii) Divergence in developmental molecular mechanisms creates different patterns of gene expression across species. For example, *MAB21L2* is required for eye morphogenesis and cell survival in the developing optic cup and lens in humans yet *mab21l2* expression patterns in the chick, mouse and zebrafish are still unknown. ^5^^–^^7^ (iii) Mature anatomy of the eye can vary between species, such as the absence of the macula in rodents and other small mammals. ^8^ (iv) Disease phenotypes observed in animals do not always mimic those seen in humans. For instance, large phenotypic variability has been observed between Usher syndrome patients and mouse models in relation to vision loss. ^9^ Embryonic lethality described in many animal models (e.g., *Sox2*, *Otx2*, and *Mab21l2* mutations in mice) is also inconsistent between species, demonstrating further variation. ^10^ As a result, there is a pressing need for physiologically relevant human models to study ocular development and disease. However, investigating mechanisms of early ocular malformations using human samples is impractical due to the inaccessibility of foetal tissue from 4 to 7 weeks of gestation. ^11^ Consequently, the use of human induced pluripotent stem cells (hiPSCs) is an attractive option to overcome these difficulties.

hiPSCs are generated from somatic cells by delivery of the ‘Yamanaka’ factors, *OCT4, SOX2, KLF-4* and *C/L-MYC.* ^12^^,^^13^ Overexpression of these transcription factors activates endogenous gene expression and reverts cells to a pluripotent state. ^14^ These pluripotent cells can then be differentiated into any cell type of interest, to study their cellular and genetic properties. While it is still unknown to what extent hiPSCs can entirely replace human embryonic stem cells (hESCs) due to the unique epigenetic signature contained in each individual line, it is important to note the distinct advantages of hiPSCs over hESCs. hiPSC use circumvents the ethical concerns associated with the creation of hESC lines from embryos as they are generated from somatic cells such as blood, urine or skin samples. ^15^ Additionally, cell lines can be generated directly from patients, creating a wide range of applications including *in vitro* disease modelling to further understanding of mutation-specific disease pathophysiology and providing targets for novel therapeutic development, while also facilitating a personalised approach as the reprogrammed cells retain all original somatic mutations. ^16^^,^^17^ In patients with a confirmed genetic diagnosis, CRISPR/Cas9 gene editing can be used to correct the mutation in their specific hiPSC line to demonstrate phenotypic recovery. ^18^^,^^19^ Gene editing can also be used to introduce a known mutation into wild type hiPSCs where patient cells are not available to create disease models with isogenic controls. ^20^

This technology has been harnessed to generate self-organising *in vitro* optic cups which recapitulate ocular development long-term, initially relying on the spontaneous differentiation of EBs to neuroepithelium followed by intrinsic cellular cues driven by cell culture conditions modulating developmental signalling pathways such as Wnt and Notch. ^21^ Three-dimensional differentiation protocols are advantageous as they closely recapitulate retinal microarchitecture, generate a high percentage of retinal cells, and facilitate self-organization to mature ocular tissue with high fidelity to human eye development. ^3^^,^^22^ Pluripotent stem cell-derived organoids have become widely used for modelling eye development *in vitro* through the generation of long-term self-organising retinal organoids. ^21^^,^^23^ Accordingly, retinal organoids grown for upwards of 25 weeks have been used to model late-onset disorders such as Leber Congenital Amaurosis, retinitis pigmentosa and *CRB1*-associated retinal dystrophy affecting photoreceptor function. ^24^^–^^27^ These disease models have advanced therapeutics development through antisense oligonucleotides, adeno-associated virus gene delivery and CRIPSR/Cas9 correction of genetic mutations to rescue disease phenotypes *in vitro.* ^25^^–^^27^ However, there has been limited modelling of developmental eye disorders at earlier timepoints, partially due to the difficulty to recapitulate *in vitro* the interactions of the invaginating optic cup and surface ectoderm, with only microphthalmia and congenital hereditary endothelial dystrophy investigated using hiPSC-derived models at less than five weeks differentiation. ^3^^,^^28^^–^^31^ The combination of limited knowledge of early ocular development and disease, inaccessibility of human foetal tissue from early developmental time points and lack of replicable cellular models suggests a need to develop a robust protocol faithful to human ocular development to further investigate early eye disorders. Here we describe our highly reproducible modified protocol that can consistently generate hiPSC-derived early optic vesicles to investigate early human ocular development and disease.

In this study, we present an adapted protocol to generate optic vesicles that can recapitulate early ocular development *in vitro.* ^32^ Originally, embryoid bodies (EBs) are generated from hiPSCs to undergo retinal differentiation. ^32^^,^^33^ A consequent study showed mechanical dissociation of hiPSCs and stable cell culture conditions generated the optimal EBs for retinal differentiation but this method does not control for EB diameter. ^22^ Cowan *et al.* investigated the relationship between EB diameter and the efficiency of retinal differentiation to generate optic cups, suggesting a diameter of roughly 275 μm for optimal retinal differentiation. ^34^ Here the use of the Aggrewell ^®^ plate allows for stringent control of EB size for optimal retinal differentiation.

Additionally, we present an alternative to animal models by describing an efficient adapted protocol for the generation of stem cell-derived optic vesicle-like models to elucidate key pathways regulating early eye morphogenesis and identify molecular disruptions underlying developmental eye disorders such as microphthalmia, anophthalmia and aniridia that arise in the first six weeks of ocular development. This protocol will help developmental biologists and geneticists working with animals consider replacement with a more faithful and physiologically relevant model of human eye development. This shift will significantly reduce the use of animals in the study of ocular development and disease.

## Methods

### Ethics and consent

This study falls under ethics 11/LO/243 NRES study of congenital eye disease under the National Research Ethics Service from Moorfields Eye Hospital NHS Foundation Trust.

Written informed consent for publication of the participants’ details and/or their images was obtained from the participants.

### hiPSC derivation

hiPSCs were derived from human dermal fibroblasts obtained from healthy male volunteers aged 23, 28 and 39 of varying ethnicities and characterised for pluripotency markers and absence of chromosomal anomalies, as described in detail in Méjécase *et al.* 2020. ^35^ In brief, fibroblasts were derived from skin biopsies after overnight incubation in digestion media (DMEM high glucose with pyruvate/glutamine (ThermoFisher Scientific, USA, cat#11995073), 20% fetal bovine serum (FBS) (ThermoFisher Scientific, USA, cat#26140079), 0.25% Collagenase I (ThermoFisher Scientific, USA, cat#17100017), 0.05% DNase I (ThermoFisher Scientific, USA, cat#EN0521), 1% penicillin/streptomycin (P/S) (ThermoFisher Scientific, USA, cat#15070063)), followed by culture in derivation media (DMEM, 20% FBS, 1% penicillin/streptomycin) and passaged using TrypLE Express (ThermoFisher Scientific, USA, cat#12605010). 1×10 ^6^ fibroblast cells were electroporated (1600 V, 20ms, 3 pulses) with 1 μg each of four episomal plasmids (pCXLE-hSK (Addgene ID# 27078), pCXLE-hUL (Addgene ID# 27080), pCXLE-hOCT3/4-shp53-F (Addgene ID# 27077) and pCXWB-EBNA1 (Addgene ID# 37624)) using the Neon Transfection System. ^25^ Transfected cells were plated on 0.1% gelatin-coated 100 mm dishes in fibroblast media with 0.5 mM sodium butyrate (Sigma-Aldrich, cat#B5587). After seven days, cells were dissociated with TrypLE Express and 200,000 cells plated into each well of a Matrigel-coated (Corning, USA, cat#354377) 6-well plate in mTeSR Plus (STEMCELL Technologies, Canada, cat#1000276). Rudimentary hiPSC colonies were excised from these plates and cultured in isolation. iPSCs were maintained in mTeSR Plus and passaged using ReleSR (STEMCELL Technologies, Canada, cat#05872).

### Embryoid body formation and measurement

EBs were formed in Aggrewell™ plates, a plate where each well of a 24-well plate is comprised of 1200 microwells, as per manufacturer’s instructions (STEMCELL Technologies, Canada, cat#34415). Briefly, hiPSCs were washed with PBS and detached with Accumax (ThermoFisher Scientific, USA, cat#00-4666-56) to form a single cell suspension. After 5-8 minutes, mTeSR Plus media was added to each well. Cells were counted using Countess™ II Automated Cell Counter (ThermoFisher Scientific, USA). 3.6 × 10 ^6^ cells per well were centrifuged and resuspended in 1mL mTeSR Plus with 10 μM Y-27632 and added to one well of the Aggrewell™ plate (3000 cells per microwell). Mixing with a pipette was required to ensure uniform distribution of cells. The plate was spun at 100 × g for three minutes and incubated at 37°C. After 24 hours, 1mL media was changed (day 1).

To measure EB diameter, the diameters of 60 individual EBs from three independent rounds of EB formation were measured using ImageJ (NIH, USA). Initially, EB diameters were measured in pixels using the ImageJ software by drawing a line across each EB and using the ‘Measure’ function. Lengths in pixels were converted to micrometres based on the manufacturers’ data. For the 2× objective, the pixel size was 3.0854 μm/pixel; for the 4× objective it was 1.5427 μm/pixel and for the 10x objective it was 0.6172 μm/pixel. These ratios allowed EB diameters to be calculated from light microscopy images.

### Optic vesicle differentiation

**Figure 1.**
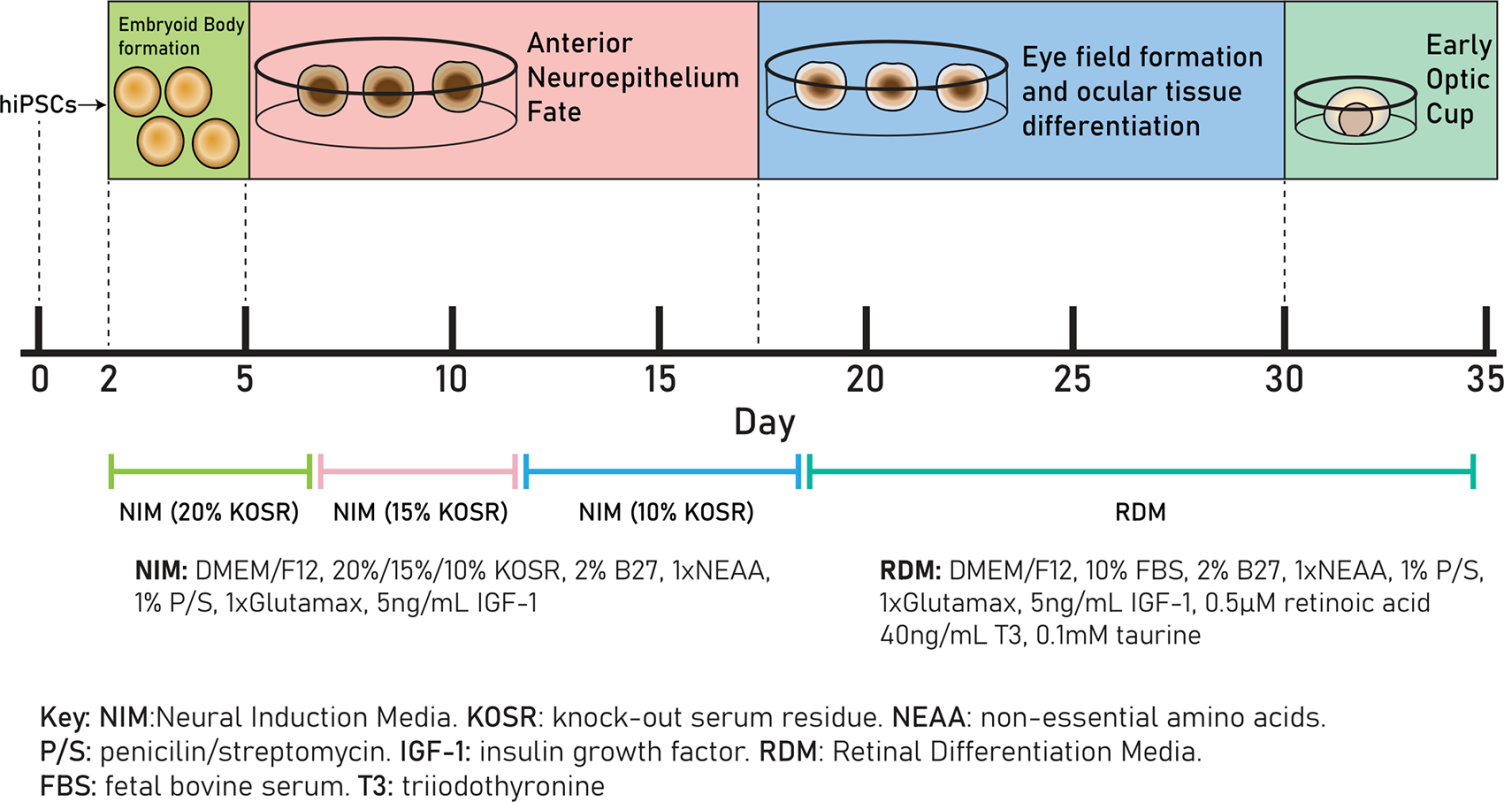
Schematic of retinal differentiation protocol from day 0 – day 35. Cells are cultured in neural induction media (NIM) (20% KOSR) from day 0 to day 7, in NIM (15% KOSR) from day 7 to day 11, in NIM (10% KOSR) from day 11 to day 18 and in retinal differentiation media (RDM) from day 18 to day 35.

Differentiation was performed as outlined by Mellough *et al.* in 2015 and Chichagova *et al.* in 2019 ^32^^,^^33^ ( Figure 1). 48 hours after EB formation (day 2), EBs were plated by gentle pipetting into 60mm TC-treated culture dishes (Appleton Woods, UK, cat#BF152) and cultured in Neural Induction Media (NIM), (DMEM/F12 (ThermoFisher Scientific, cat#31331028), 20% knock-out serum residue (KOSR) (ThermoFisher Scientific, cat#10828028, 2% B27 (ThermoFisher Scientific, USA, cat#17504001), 1xnon-essential amino acids (NEAA; ThermoFisher Scientific, USA, cat#11140050), 1% P/S, 1xGlutamax (ThermoFisher Scientific, USA, cat#35050061) and 5 ng/mL IGF-1 (Sigma-Aldrich, USA, cat#I3769). One well of an Aggrewell™ plate was transferred into six uncoated 60mm round dishes, resulting in a final density of approximately 200 EBs per 60mm culture dish, or 1200 EBs per 3.6 × 10 ^6^ cells.

Cells were cultured in NIM with decreasing KOSR concentrations, 20% from day 2-7, 15% from day 7-11 and 10% from day 11-18. From day 18, cells were cultured in Retinal Differentiation Media (DMEM/F12, 10% FBS, 2% B27, 1×NEAA, 1×Glutamax, 1% P/S, 5 ng/mL IGF-1, 0.1 mM taurine (Sigma-Aldrich, USA, cat#T8691), 40 ng/mL triiodothyronine (Sigma-Aldrich, USA, cat#T6397) and 0.5 μM retinoic acid (Sigma-Aldrich, USA, cat#R2625) added immediately before use. Cells were cultured in RDM until day 50. Optic vesicle diameters were measured as described above.

### RT-qPCR

Each individual plate of optic vesicles was collected at either day 0, day 20 or day 35 and RNA extraction was performed using the RNeasy Mini Kit (QIAGEN, Germany, cat#74104). cDNA was synthesized from 1μg using the SuperScript III First Strand cDNA synthesis kit (Invitrogen, USA, cat#18080093) according to manufacturer’s instructions. RT-qPCR was performed using 2× SYBR Green Master Mix (ThermoFisher Scientific, USA, cat#4472908) as per manufacturer’s instructions on the StepOne Real-Time PCR system (Applied Biosystems, ThermoFisher, UK). Primers used for the qRT-PCR at 200 nM are listed in Table 1 and were designed using the Primer-BLAST tool from the National Centre for Biotechnology Information. All transcript levels were measured in triplicate and normalised to *GAPDH*, with undetermined C _T_ values in negative controls where no cDNA was present. The relative expression of each target gene compared to iPSCs at day 0 of differentiation was calculated using the comparative C _T_ method. ^36^ Statistical comparisons were performed with Microsoft Excel (Microsoft, USA) and figures generated using GraphPad Prism (GraphPad Software, USA) or Microsoft Excel (Microsoft, USA).

**Table 1.** qRT-PCR primers used in optic vesicle characterisation.

Primers		
	Target	Forward/Reverse primer (5′-3′)
House-Keeping Genes (qRT-PCR)	*GAPDH*	ACAGTTGCCATGTAGACC/TTTTTGGTTGAGCACAGG
Early Ocular Marker Genes	*PAX6*	GGCCGAACAGACACAGCCCTCAC/ATCATAACTCCGCCCATTCACC
	*RAX*	AGGCGGAAAAATAGAGTTTG/TACCCCAATTATTCACTCCTC
	*OTX2*	TAAAAATTGCTAGAGCAGCC/CATGGGAGGTTAGAAAAAGTC
	*VSX2*	GGCGACACAGGACAATCTTTA/TTCCGGCAGCTCCGTTTTC
	*MITF*	CAGTACCTTTCTACCACTTTAG/CCTCTTTTTCACAGTTGGAG
	*SOX2*	TTCACATGTCCCAGCACTACCAGA/TCACATGTGTGAGAGGGGCAGTGTGC

### Statistics

Statistical analysis was performed using Excel (Microsoft, USA). A two-tailed unpaired Student’s t-test was used for comparison studies. A *p-*value of <0.05 was considered statistically significant. Significance levels were set when *p<*0.05 (*), *p<*0.01 (**), *p<*0.001 (***). All results are expressed as mean±SD unless specified. All experiments were performed with n=3 replicates grown at separate times in separate dishes.

A sample size of n=3 was chosen as each sample is time-, labour- and cost-intensive to generate and cannot be equated to cell lines used for high throughput experiments which are simpler and cheaper to maintain. This is commonly seen in the literature as shown in other studies investigating ocular development and disease, with some only using up to three clonal hiPSC lines per condition. ^22^^,^^25^^,^^28^^,^^37^ Consequently, we chose a sample size of n=3 to satisfy these concerns.

### Embedding and cryoembedding of vesicles

Optic vesicles were fixed in 4% paraformaldehyde (Fisher Scientific, UK, cat#10532955 for 10-20 minutes at 4°C, washed three times with PBS and stored overnight at 4°C in 30% sucrose (Sigma-Aldrich, USA, cat#S0389). Vesicles were embedded individually in 1.5 cm × 1.5 cm × 0.5 cm moulds (Fisher Scientific, UK, cat#11670990) containing 800 μL PBS, 7.5% gelatin and 10% sucrose (Sigma-Aldrich, USA cat#G2500) solution and left to solidify at 4°C overnight. Embedded vesicles were excised from their moulds and placed in OCT embedding media (Agar Scientific, UK, cat#AGR1180) prior to snap-freezing in -50°C 2-methylbutane using a small dewar in a fumehood (Sigma-Aldrich, USA, cat#M32631) for three minutes. Frozen blocks were stored at -80°C.

### Immunohistochemistry

Vesicles were sectioned using the Leica CM 3050 S cryostat at a thickness of 10 μm and slides were left at RT for 1-2 hrs. Slides were washed twice for five minutes in PBS+0.1%Tween ^®^20 (PBS/T) (Sigma-Aldrich, USA, cat#P1379) and permeabilized in PBS/T+0.5% Triton-X (Fisher Scientific, UK, cat#10591461) for one hour at RT with slight agitation. Samples were washed with PBS/T for five minutes and then blocked for one hour at RT in PBS+0.2% gelatin+0.5% Triton-X. Samples were incubated with primary antibodies overnight at 4°C (primary antibodies and dilutions can be found in Table 2). Serial sections were used as not all primary antibodies could be incubated together as they came from the same host animal. For day 20 sections, serial sections were incubated alone with mouse anti-RAX followed by goat anti-OTX2 and rabbit anti-PAX6 for clearer imaging with the confocal microscope. At day 35, serial sections were incubated with either mouse anti-VSX2 and rabbit anti-PAX6 or mouse anti-SOX2 to avoid mouse anti-VSX2 and anti-SOX2 incubation on the same slide. At day 50, serial sections were incubated with mouse anti-CRX and rabbit anti-PAX6, followed by mouse anti-VSX2 and rabbit anti-BRN3B, followed by goat anti-OTX2 and rabbit anti-RECOVERIN. Samples were subsequently washed three times for ten minutes with PBS/T. Samples were then incubated with secondary antibodies for one hour at RT in the dark (secondary antibodies and dilutions can be found in Table 2). Samples were washed again three times for five minutes with PBS/T and once for five minutes with PBS. Slides were dipped in 100% ethanol and left to dry at RT. Once dry, coverslips were mounted with ProLong™ Diamond Antifade Mountant with DAPI (ThermoFisher Scientific, USA, cat#P36971) and left to set overnight at RT in the dark. Slides were imaged using the confocal microscopes ZEISS LSM 700 and LSM 710 (ZEISS Research, Germany) and figures were generated using ImageJ (NCBI, USA) and Adobe Illustrator (Adobe Inc, USA).

**Table 2.** Primary and secondary antibodies used for optic vesicle validation.

Antibodies			
	Antibody	Dilution	Company Cat # and RRID
Primary antibodies (Optic vesicle markers)	Goat anti-OTX2	1:75	R and D Systems cat#AF1979, RRID: AB_2157172
	Rabbit anti-PAX6	1:100	Covance Cat# PRB-278P, RRID: AB_291612
	Mouse anti-RAX	1:200	Insight Biotechnology cat#sc271889, RRID: AB_10708730
	Mouse anti-VSX2	1:200	Santa Cruz Biotechnology cat#sc-365519, RRID: AB_10842442
	Mouse anti-SOX2	1:50	Santa Cruz Biotechnology cat#sc-365823, RRID: AB_10842165
	Mouse anti-CRX	1:500	Abnova cat#H00001406-A01, RRID: AB_462432
	Rabbit anti-Recoverin	1:500	Merck Millipore cat#AB5585, RRID: AB_2253622
	Rabbit anti-BRN3B	1:300	Abcam cat#ab56026, RRID: AB_880587
Secondary antibodies	Goat anti-Mouse IgG (H+L) Cross-Adsorbed Secondary Antibody, Alexa Fluor 647	1:800	Thermo Fisher Scientific Cat# A-21235, RRID:AB_2535804
	Goat anti-Rabbit IgG (H+L) Highly Cross-Adsorbed Secondary Antibody, Alexa Fluor 488	1:800	Thermo Fisher Scientific Cat# A32731, RRID:AB_2633280
	Goat anti-Mouse IgG (H+L) Cross-Adsorbed Secondary Antibody, Alexa Fluor 488	1:800	Thermo Fisher Scientific Cat# A-10011, RRID:AB_2534069
	Donkey anti-Goat IgG (H+L) Cross-Adsorbed Secondary Antibody, Alexa Fluor 647	1:800	ThermoFisher Scientific Cat# A-21447, RRID:AB_2535864
	Donkey anti-Goat IgG (H+L) Cross-Adsorbed Secondary Antibody, Alexa Fluor 488	1:800	ThermoFisher Scientific Cat# A-32814, RRID:AB_2762838

## Results

### Optic vesicle characterisation

**Figure 2.**
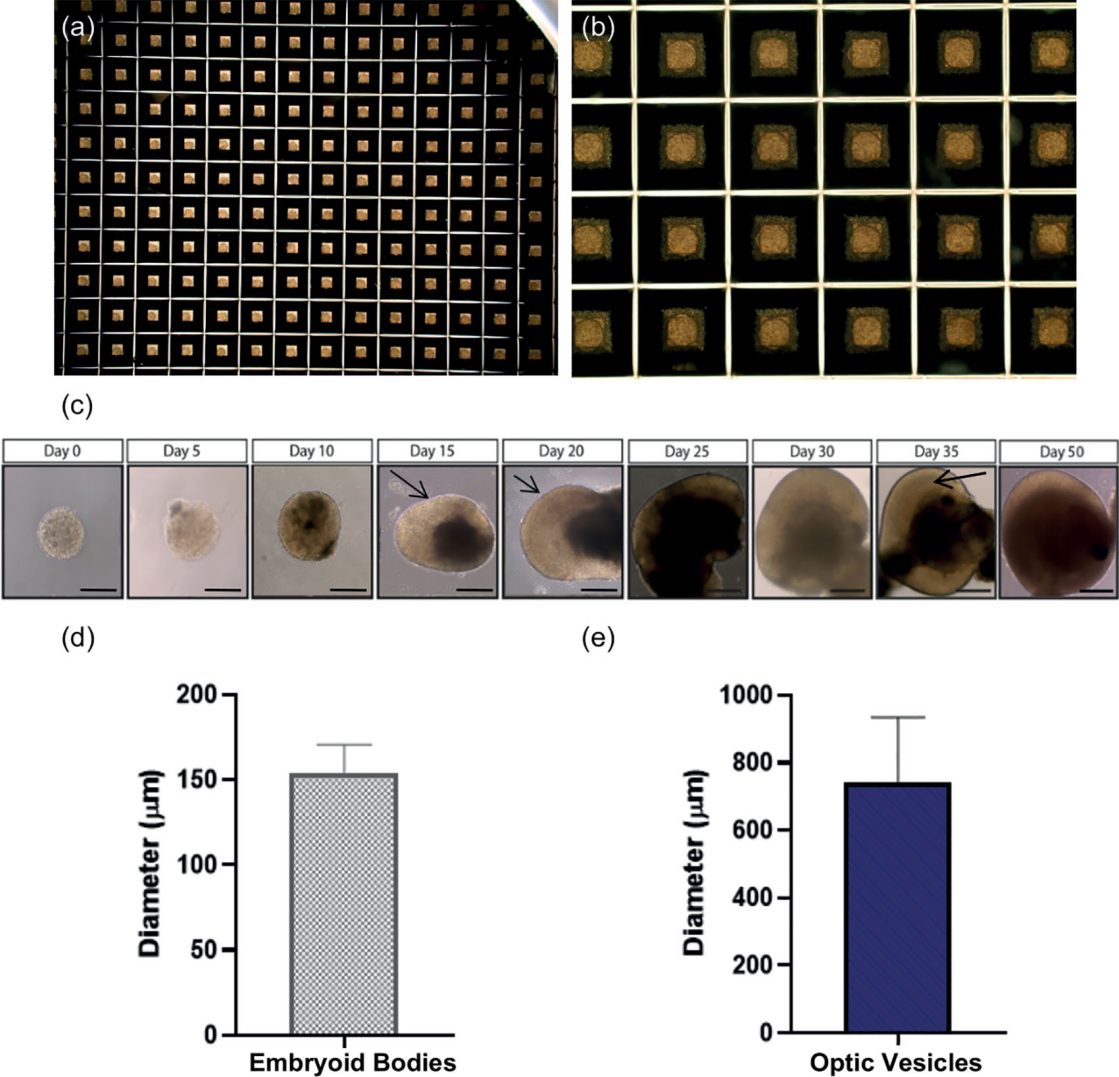
Embryoid bodies formed in Aggrewell ™ plates. (a-b) Uniform embryoid bodies formed in Aggrewell ™ plates after two days culture in mTeSR Plus with Y-27632 photographed at 2× and 10× magnification. (c) Brightfield images of differentiating optic vesicles from embryoid bodies at day 2 to laminated optic vesicles at day 35. At day 15, arrows indicate developing neuroepithelium. At day 20, arrows indicate initial lamination detected in optic vesicles. At day 35, arrows indicate complete laminar neuroepithelium comprised of retinal progenitor cells. Scale bar represents 150 μm. (d) Average diameter of embryoid bodies formed at day 2 (n=3 replicate rounds of 60 embryoid bodies). Error bars represent standard deviation. (e) Average diameter of optic vesicles measured at day 35. (n=3 replicate rounds of 60 embryoid bodies). Error bars represent standard deviation.

Optic vesicles were easily identifiable due to their distinct morphology, namely laminated neuroepithelium appearing like a thick ribbon in the outer layers of the developing optic vesicle, that was observable from ~day 20 using light microscopy ( Figure 2). To characterise optic vesicles, we measured mRNA levels of known markers of early eye formation at day 20 and day 35 of differentiation as these timepoints corresponded to optic vesicle and cup formation respectively and performed immunohistochemistry at day 20, 35 and 50. ^28^^,^^29^ By confirming expression of ocular development markers, we aimed to validate the retinal fate of our optic vesicle models.

### Optic vesicle-like models display distinct morphological changes by day 20/35

By day 2, uniform EBs had formed in Aggrewell ^®^ plates ( Figure 2a-b). By day 5, EBs became visibly denser due to cell proliferation. By day 20, limited self-organisation and lamination was observed through a phase-bright layer at the edge of the structure, and by day 35, complete lamination along with a cup-like sheet of neuroepithelium was observed ( Figure 2c). The average diameter of EBs at day 2 was 154.26±16.43 μm ( Figure 2d). However, the average diameter of optic vesicles at day 35 was 741.9±192.7 μm indicative of larger variation in vesicle size as differentiation progresses ( Figure 2e).

### Upregulation of early eye transcription factor mRNA is detected at day 20/35 by RT-qPCR, showing differentiation of cells to optic vesicles

**Figure 3.**
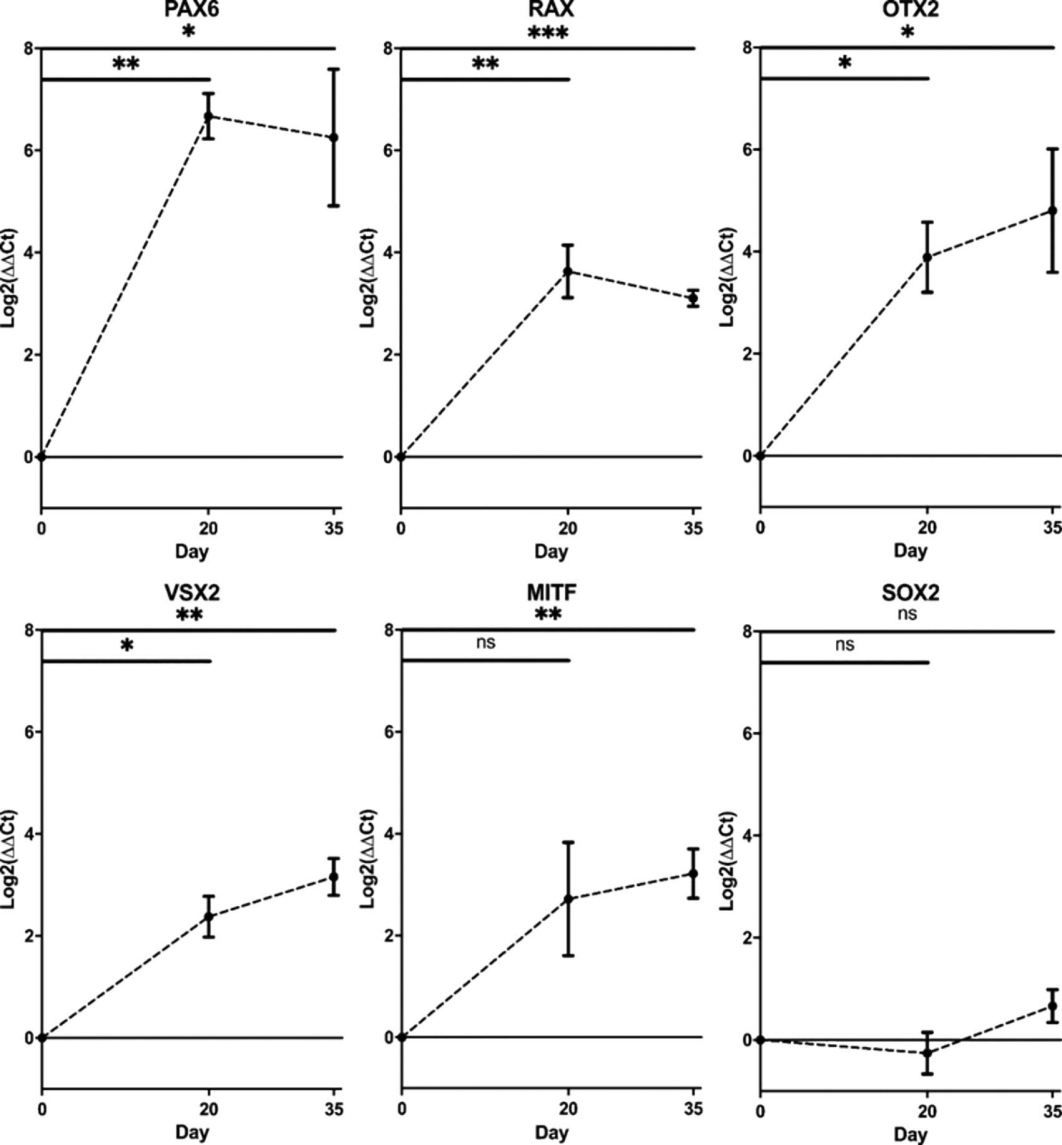
Changes in gene expression of early eye development transcription factors. *PAX6, RAX, OTX2, VSX2, MITF* and *SOX2* transcript levels in optic vesicles at day 0, day 20 and day 35. Transcript levels were measured using RT-qPCR and presented as a log _2_ fold change in expression from undifferentiated cells at day 0. Expression levels normalised to housekeeping gene glyceraldehyde 3-phosphate dehydrogenase (GAPDH). (* *p*<0.05, ** *p*<0.01, *** *p*<0.001). Error bars represent the standard deviation between replicates (n=3).

RT-qPCR demonstrated increased mRNA transcript levels of early EFTFs at day 20 compared to day 0 iPSCs, with 100-fold increase of *PAX6* (Student’s t-test, *p*<0.01) and over 10-fold increase in *RAX* (Student’s t-test *, p*<0.01) *,* alongside initial increase in retinal progenitor markers *VSX2* (Student’s t-test, *p*<0.05) and *OTX2* (Student’s t-test *, p*<0.05) ( Figure 3). By day 35, *VSX2* expression had increased 10-fold ( *p*<0.01) and there was also 10-fold upregulation of RPE marker MITF (Student’s t-test *, p*<0.01). *SOX2* expression remained constant across the differentiation (Student’s t-test *, p*>0.05), as expected given SOX2 is a marker for pluripotency, as well as early ocular development ( Figure 3). All error bars on the figure represent standard deviation between n=3 rounds of differentiation.

### Optic vesicle structure visualised by immunostaining of early ocular differentiation markers at day 20/35

**Figure 4.**
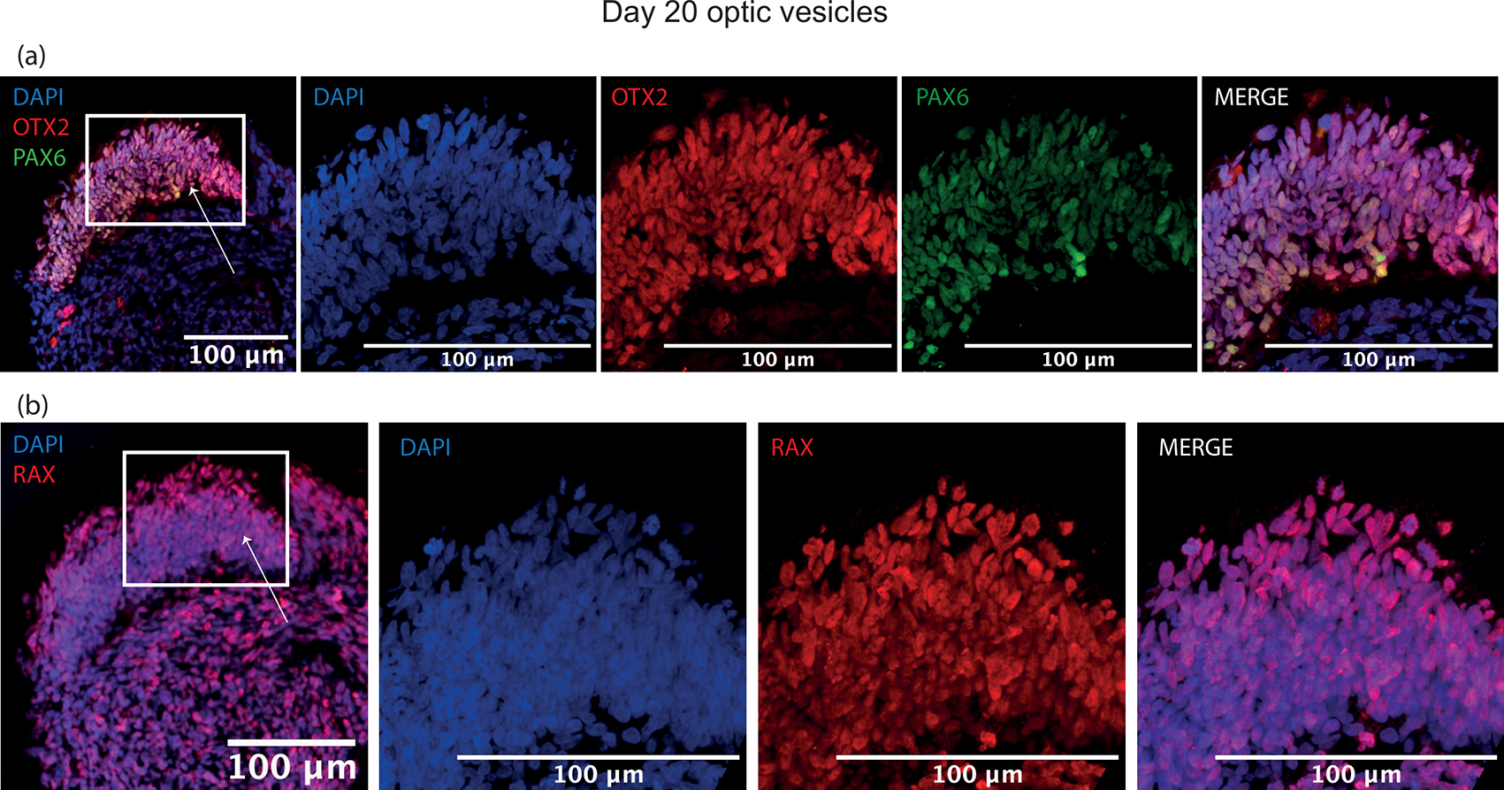
Representative images of immunohistochemistry sections of differentiating optic vesicles at day 20. Expression of early eye-field transcription factors (a) OTX2, PAX6 and (b) RAX is expressed in neuroepithelium at day 20 in optic vesicles. Arrows indicate neuroepithelial layer seen in zoomed panels.

**Figure 5.**
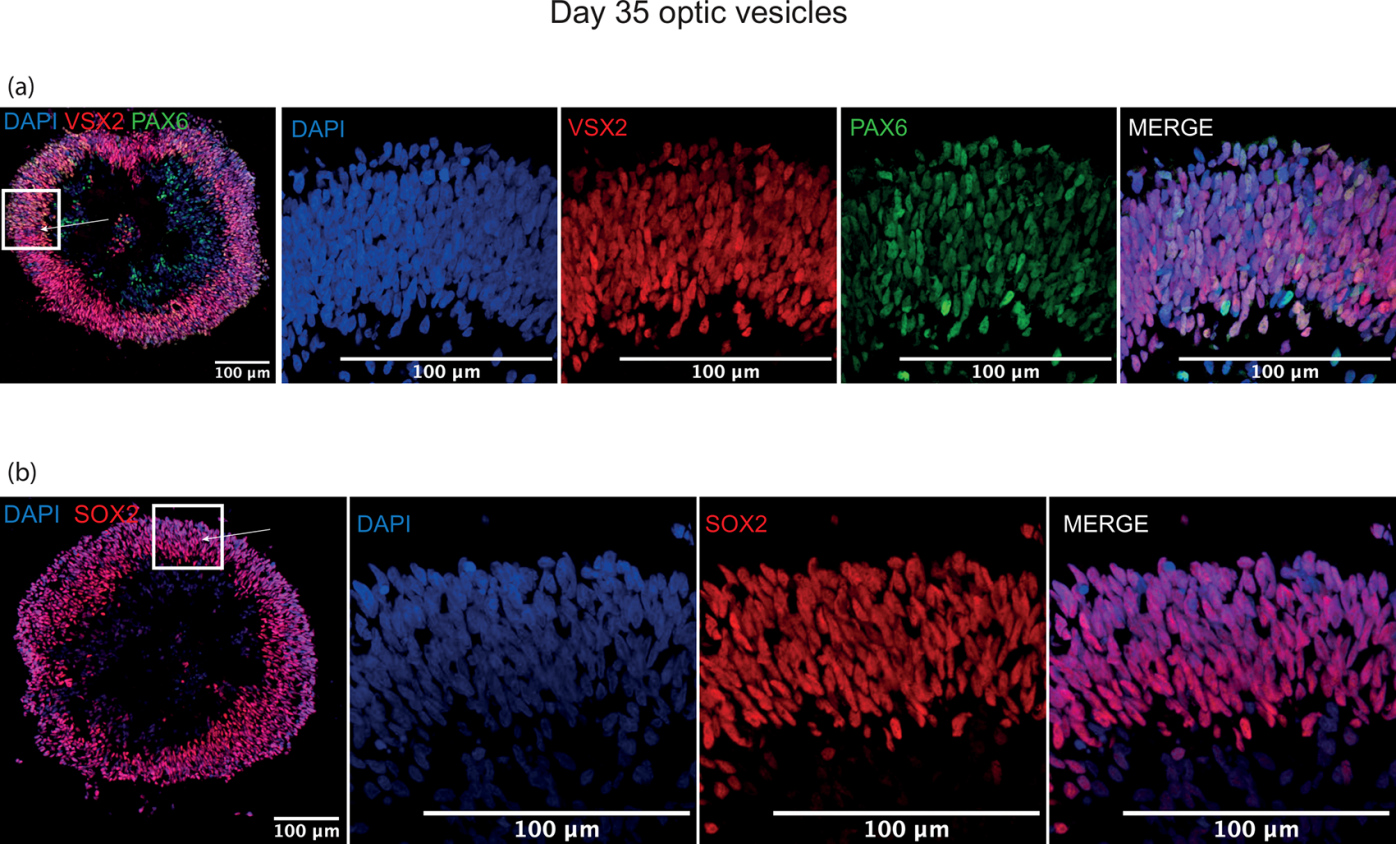
Representative images of immunohistochemistry sections of differentiating optic vesicles at day 35. Thick cellular layers of neuroepithelium are present in optic vesicles at day 35 expressing (a) early retinal progenitor marker VSX2 co-expressed with ocular development master regulator PAX6, and (b) retinal progenitor marker SOX2. Arrows indicate neuroepithelial layer seen in zoomed panels.

By day 20, a thick laminar layer of neuroepithelium was observed at the edge the developing optic vesicle expressing optic vesicle markers RAX, PAX6 and OTX2 ( Figure 4). These markers were selected as early eye-field transcription factors expressed in the developing optic vesicle. At day 35, the developing neuroepithelium had maintained its thick laminar structure and was entirely comprised of VSX2+, PAX6+ and SOX2+ cells, indicative of a retinal progenitor fate ( Figure 5). These markers were selected to show a commitment to an early retinal fate.

### Emergence of mature neural retina cell types demonstrated by protein expression

**Figure 6.**
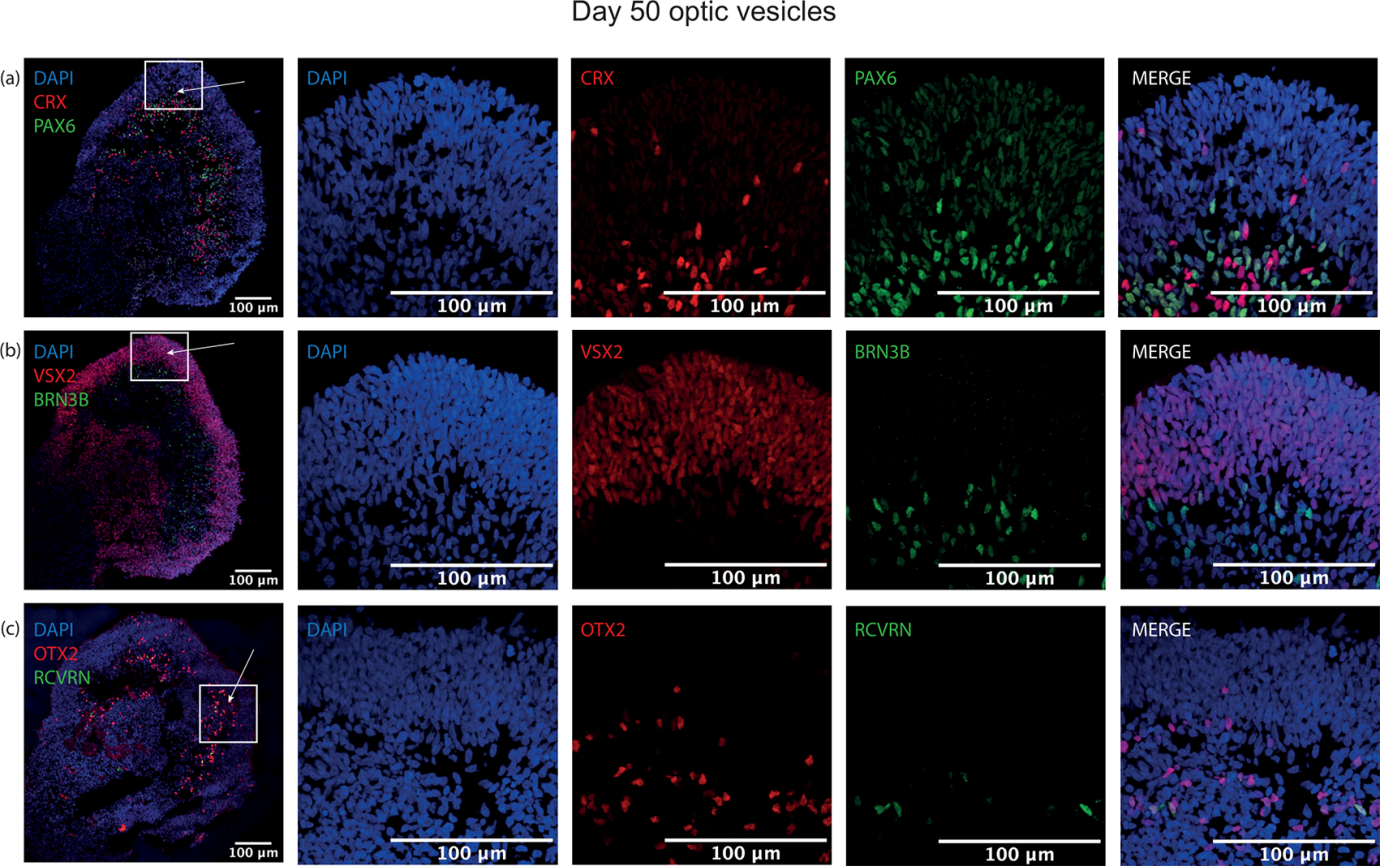
Representative images of immunohistochemistry sections of differentiating optic vesicles at day 50. Laminar neuroepithelium thickens at day 50, with differentiating photoreceptor precursor cells detected by (a) CRX expression strongest at the basal aspect but present in the neuroepithelium characterised by retinal progenitor cells expressing (b) VSX2. Retinal ganglion cell marker (b) BRN3B is expressed closer to the centre of the optic vesicle rather than in the neuroepithelial cell layer. (a) PAX6 is expressed in the neuroepithelium and towards its basal aspect, where (c) few OTX2+ cells co-localise with early rod marker Recoverin (RCVRN) also indicative of photoreceptor precursor cell differentiation.

To ascertain whether this protocol could generate more mature retinal-specific cell types, optic vesicles were cultured to day 50. At this point, retinal progenitor marker VSX2 was maintained with a similar laminar expression to day 35 at the outer layer of the structure ( Figure 6). Clear expression of the photoreceptor progenitor marker CRX was detected, with strongest signal in the basal aspect of the differentiating neuroepithelial layer, co-localising with PAX6 ( Figure 6). At this timepoint, ubiquitous retinal progenitor marker OTX2 was also observed throughout the structure, with some OTX2+ cells expressing with rod precursor marker Recoverin, further indicating photoreceptor specification ( Figure 6). Additionally, retinal ganglion cell progenitor marker expression BRN3B was detected at the basal aspect of the neuroepithelium, demonstrating further lamination of the neural retina and the emergence of retinal ganglion cells ( Figure 6).

## Discussion

In this study, we report the generation of *in vitro* optic vesicles using a protocol adapted from Mellough *et al.* and Chichagova *et al.* ^32^^,^^33^ We describe distinct morphological changes displayed by developing optic vesicles by day 35 and the expression of early ocular markers PAX6 *,* RAX, OTX2, VSX2, MITF and SOX2 measured by RT-qPCR and immunohistochemistry. The detection and expression patterns of these markers highlight extensive laminar formation by day 35, as described in previous studies, suggesting high replicability of this protocol to generate hiPSC-derived ocular vesicles faithful to human eye development. ^22^^,^^32^^,^^38^^–^^40^ Importantly, we detect the expression of more mature photoreceptor and retinal ganglion cell progenitor markers such as CRX, OTX2 and BRN3b by day 50, suggesting this protocol can generate retinal lineage-specific organoids. Similar expression patterns have been detected in human foetal tissue (HFT), suggesting a high fidelity between *in vitro* optic vesicles and HFT. ^40^^,^^41^

Capowski *et al.* suggest the emergence of laminar morphology in differentiating vesicles, as observed in our study, is a key tool to identifying retinal organoids at approximately day 35. ^42^ Additionally, those structures ubiquitously expressed early ocular markers such as VSX2 suggesting a close link between morphological and molecular changes during early optic vesicle differentiation. ^42^ For long-term studies, the identification of mature cell types and retinal microarchitecture using immunohistochemistry would validate the efficacy of this protocol to generate rod and cone photoreceptors, retinal ganglion, horizontal and bipolar cells complemented with Müller glia organised into a laminar structure that contains three neuronal cells layers connected by two plexiform layers. ^43^ This striking cellular structure of a human retina *in vivo* is recapitulated in retinal organoids and is indicative of mature retinal cell formation. Electroretinography, which provides an important functional readout for photoreceptors and retinal ganglion cells similar to the human retina, can be measured *in vitro* or *in vivo* upon transplantation into animal models. ^44^^,^^45^

Previous studies reported that an EB diameter of roughly 275 μm as well as enzymatic rather than mechanical passage of hiPSCs prior to EB formation are optimal for most efficient optic vesicle differentiation. ^22^^,^^34^ Our study has shown differentiation of EBs roughly 160 μm in diameter, suggesting that the optimal EB diameter for retinal differentiation may differ between cell lines. The standardised generation of EBs of similar size is particularly difficult yet the low variance in diameter we observed suggests our protocol has improved the standardisation of EB size optimal for differentiation to optic vesicles. Although the use of Aggrewell ^®^ plates can standardize the size of EBs in the beginning of the process, it is much more difficult to control the size of actual vesicles differentiating in culture. This is not something we successfully achieved in this study, nor has this been documented in previous studies. Additionally, our data shows large variability in vesicle diameter at day 35, with a range of 383.80 μm-1119.29 μm reported in vesicles derived from healthy controls. Furthermore, in our study, the standard deviation in vesicle diameter at day 35 was 192.7 μm which did not significantly differ from standard deviations reported in vesicle diameter by Guo *et al.*, suggesting vesicle size is difficult to regulate *in vitro* independently of the differentiation protocol. ^46^ This is significant as vesicle size can impede a response to therapeutics. ^47^ Novel improvements to protocols that will standardize vesicle size over the timecourse of differentiation will enhance therapeutics testing in these models and reduce variability between samples and groups.

Two-dimensional *in vitro* cellular models as well as animal models were the initial widespread platform for basic science research and pre-clinical therapy testing. However, the lack of a three-dimensional microenvironment and mechanical cues to guide differentiation, and the aforementioned disadvantages of animal models, reduce the relevance of these models to human disease. ^48^ Although here we have described the generation of *in vitro* optic vesicles to model early ocular development and disease, the original protocol has generated complex photoreceptor-like cells differentiated for upwards of 22 weeks, thus can efficiently be expanded for more complex modelling of later stages of eye development and later-onset retinal diseases. ^33^^,^^49^

A considerable disadvantage to hiPSC-derived optic vesicle generation is the large variability observed both between differentiation of different hiPSC lines and also between rounds of differentiation of the same hiPSC clone; thus many lines should be screened to ascertain their differentiation capacity. ^3^^,^^34^ Biomarkers predicting the differentiation efficiency are particularly useful such as elevated levels of epigenetic marker H3K4me2 or downregulated *Meis1* transcript levels. ^50^ However, pre-screening methods are labour intensive and extremely costly, particularly following the expense, time and expertise already required for hiPSC reprogramming and characterisation. ^34^ Therefore, further technical optimisations are required to increase differentiation efficiency as well as complexity of these model systems.

Nonetheless, the use of hiPSC-derived optic vesicles and retinal organoids can greatly reduce animal use when investigating ocular development and disease. In 2019/20, approximately ~75 original research papers were published using iPSC models of ocular development or disease. This is much lower in comparison to the ~750 original research papers still using animal models. The uptake of this method amongst animal researchers would greatly increase the number of iPSC-based papers while simultaneously effectively reducing animal model experimentation due to the greater accessibility of a human-derived physiologically faithful model of human ocular development. ^3^ This effect has been observed locally as our group has not created zebrafish models for early developmental disorders that have been modelled using stem-cell derived optic vesicles. As mentioned above, each zebrafish knockout line requires approximately 750 fish per mutation created by CRISPR/Cas9 gene editing and 250 per morpholino knockout. The method described here has greatly reduced the number of zebrafish used in our research by at least 750 fish per patient cell line. This would contribute greatly to the 3Rs aim of replacing animal models.

Further advancements to the methodology described here, such as retina-on-a-chip and retinal differentiation using bioreactors have enhanced *in vitro* differentiation to reduce variability of organoid models, and more closely recapitulate native retina physiology and the human embryological environment. ^51^^,^^52^ The combination of *in vitro* retinal tissue generated by this protocol with either a microchip containing a flow system mimicking blood flow or growth in a bioreactor more closely resembling the environment for embryonic development *in utero,* will enhance the modelling of ocular development and disease at both early and late developmental stages. Research is ongoing to create more complex *in vitro* models recapitulating the anatomy of the eye, such as a photoreceptor/RPE/choroid complex using patient derived cells.

In this study, we report an adapted protocol for the generation of hiPSC-derived optic vesicles that faithfully recapitulates early human eye development. The improved standardisation of EB generation was a key adaptation of this protocol to enhance efficiency of optic vesicle generation. Next-generation sequencing techniques and future omics studies will provide novel insights into early eye development, further understanding ocular maldevelopment that occur in diseases like microphthalmia/anophthalmia or coloboma, which currently have no treatment. This model is an exciting development for further understanding of human ocular development and disease and can be an important pre-clinical platform for the development of novel therapeutics.

## Data availability

### Underlying data

Zenodo: Efficient embryoid-based method to improve generation of optic vesicles from human induced pluripotent stem cells data, https://doi.org/10.5281/zenodo.6332896.

This project contains the following underlying data:

‐ Figure 2 Embryoid bodies n1.jpg
‐ Figure 2 Embryoid bodies n2.jpg
‐ Figure 2 Embryoid bodies n3.jpg
‐ Figure 2 Embryoid bodies zoom.jpg
‐ Figure 2 embryoid body size.xlsx
‐ Figure 2 wt optic vesicle sizes d35.xlsx
‐ Figure 3 qPCR Raw Data_12.01.22.xlsx
‐ Figure 4 a PAX6 OTX2 panel - C1-MAX_28.04.21_WT1_D20.4_031221_DAPI_630x_crop.jpg
‐ Figure 4 a PAX6 OTX2 panel - C2-MAX_28.04.21_WT1_D20.4_031221_PAX6_630x_crop.jpg
‐ Figure 4 a PAX6 OTX2 panel - C3-MAX_28.04.21_WT1_D20.4_031221_OTX2_630x_crop.jpg
‐ Figure 4b RAX panel - C1-MAX_04.05.21_WT1_D20.4_031220_DAPI_630x_crop.jpg
‐ Figure 4b RAX panel - C2-MAX_04.05.21_WT1_D20.4_031220_RAX_63x_crop.jpg
‐ Figure 5a PAX6 VSX2 panel - C1-MAX_11.05.21_WT1_D35.1_141120_DAPI_630x_crop.jpg
‐ Figure 5a PAX6 VSX2 panel - C2-MAX_11.05.21_WT1_D35.1_141120_PAX6_630x_crop.jpg
‐ Figure 5a PAX6 VSX2 panel - C3-MAX_11.05.21_WT1_D35.1_141120_VSX2_630x_crop.jpg
‐ Figure 5b SOX2 panel - C1-MAX_17.05.21_WT1_D35.1_141120_DAPI_630x_crop.jpg
‐ Figure 5b SOX2 panel - C2-MAX_17.05.21_WT1_D35.1_141120_SOX2_630x_crop.jpg
‐ Figure 6a PAX6 CRX panel - C1-MAX_04.05.21_WT1_D50.1_190421_DAPI_630x_crop.jpg
‐ Figure 6a PAX6 CRX panel - C2-MAX_04.05.21_WT1_D50.1_190421_PAX6_630x_crop.jpg
‐ Figure 6a PAX6 CRX panel - C3-MAX_04.05.21_WT1_D50.1_190421_CRX_630x_crop.jpg
‐ Figure 6b BRN3b VSX2 panel - C1-MAX_04.05.21_WT1_D50.1_190421_DAPI_630x_crop.jpg
‐ Figure 6b BRN3b VSX2 panel - C2-MAX_04.05.21_WT1_D50.1_190421_VSX2_630x_crop.jpg
‐ Figure 6b BRN3b VSX2 panel - C3-MAX_04.05.21_WT1_D50.1_190421_BRN3_630x_crop.jpg
‐ Figure 6c Recoverin OTX2 panel - C1-MAX_04.05.21_WT1_D50.1_190421_DAPI_630x_2_crop.jpg
‐ Figure 6c Recoverin OTX2 panel - C2-MAX_04.05.21_WT1_D50.1_190421_RCVN_630x_2_crop.jpg
‐ Figure 6c Recoverin OTX2 panel - C3-MAX_04.05.21_WT1_D50.1_190421_OTX2_630x_2_crop.jpg

Data are available under the terms of the Creative Commons Attribution 4.0 International license (CC-BY 4.0).
