# Supplementary material for: Efficient embryoid-based method to improve generation of optic vesicles from human induced pluripotent stem cells
Source: F1000Res. 2022 Mar 17;11:324. [Version 1] doi: 10.12688/f1000research.108829.1 (PMC9218590; doi:10.12688/f1000research.108829.1)
Supplement: Peer review history

# Peer review: Reviewer response for version 1

**DOI:** 10.5256/f1000research.120258.r141403

**Published:** 2022-06-22

**Authors:** J. Arjuna Ratnayaka

**Affiliations:** 1 Clinical and Experimental Sciences, Faculty of Medicine, University of Southampton, Southampton, UK

A methods article by Eintracht *et al* describes an embryoid-based (EB) approach to improve the generation of optic vesicles from human induced pluripotent stem cells (iPSCs).

 The introduction provides a comprehensive overview of the advantages of iPSCs vs. embryonic cells. The benefits of 3D-retinal organoid models are also summarised elegantly in the lead-up to making a compelling case for these to be used for studies of early ocular development. However, certain articles related to the development of retinal EBs have not been mentioned, some of which have also used Aggrewell plates ( https://pubmed.ncbi.nlm.nih.gov/27812821/).

 Given that this is a methods development article, the authors should seriously consider including a summary table, comparing and contrasting their improvements against published methods (Mellough *et al*, Chichagova *et al* and others). Discussion points can be linked to this table to improve clarity and highlight exactly what specific aspects of the developmental process have been enhanced by their efforts. 

**Specific comments for consideration by the authors:**

- In the introduction, the authors state that for the 2019-2021 period, ~12,000 peer-reviewed articles investigating ocular development and disease were published, with roughly 2000 articles studying ocular development and disease using animal models. Approximately half of these papers investigated early ocular development using animals that could be replaced with a human stem-cell based model. Does this imply that the remainder of the ~10,000 articles studied later stages of development and/or used *in-vitro* (non-animal) methods to obtain data? A further breakdown of this ‘umbrella’ information would be informative to provide a wider context to readers. 
- The text (in the introduction section) describing the sequence of events in their methodology can be improved for clarity. In the results section, this could be linked to the Figure 1 schematic, where the authors can indicate what types of chambers (TC-treated dishes/Aggrewells and round dishes etc.) were used at each stage of the developmental pipeline. Please see Figures 1 in Radojevic *et al* ( https://www.mdpi.com/2221-3759/9/3/38) and Hunt *et al *( https://pubmed.ncbi.nlm.nih.gov/27826002/) as examples.  

 
**Methods:**

- Immunohistochemistry: could the authors’ state how many images were collected from each antibody staining type? Please also indicate from how many individual wells and experimental repeats.

**Results: **

- Figure 2: given the findings and discussion points in this methods article, the authors should consider providing magnified brightfield images of EB formation (Figure 2C), which can help highlight the developing lamination.  
- Brightfield images for EBs are helpfully shown up to day 50. However, the authors should also consider including data for EB diameter on day 50 (only day 2 and 35 are provided). qPCR data should also be supplied for day 50 EBs. This information is important to draw comparisons with other published methods (see general comments above).
- The authors should consider providing a readout for synaptic compartments in their model by labelling pre and post-synaptic structures. As it is, there is no indication how the proposed method influences these important neuron-to-neuron connections during early retinal development. 
- The authors have stated that MITF is upregulated by day 35, indicating the presence of RPE cells in their EBs. Are the authors able to provide any RPE-specific staining in confocal images by day 35 or 50? If not, they could consider a semi-quantitative method of analysis using brightfield images of EBs.    

**Discussion: **

- In the discussion, the authors mention the importance of electroretinography measurements utilizing *in-vitro* systems and upon transplantation into animal models. Given their model is proposed as a candidate tool to assess early retinal developmental deficits, they should also consider commenting on whether electrical activity can be recoded in their model, and any limitations to such approaches. This point can demonstrate the versatility of their work to the field of retinal developmental biology, which, as they have so elegantly argued in the introduction, is lacking effective investigative tools.

Is the rationale for developing the new method (or application) clearly explained?

Yes

Are the 3Rs implications of the work described accurately?

Yes

Is the description of the method technically sound?

Yes

If applicable, is the statistical analysis and its interpretation appropriate?

Yes

Are the conclusions about the method and its performance adequately supported by the findings presented in the article?

Yes

If any results are presented, are all the source data underlying the results available to ensure full reproducibility?

Yes

Are a suitable application and appropriate end-users identified?

Yes

Are sufficient details provided to allow replication of the method development and its use by others?

Yes

Reviewer Expertise:

Retinal degeneration and links with the ageing brain, in-vitro cell and mouse models, imaging. Referee suggested by the NC3Rs for their scientific expertise and experience in assessing 3Rs impact.

I confirm that I have read this submission and believe that I have an appropriate level of expertise to confirm that it is of an acceptable scientific standard.

## References

[1] : Early retinal differentiation of human pluripotent stem cells in microwell suspension cultures. *Biotechnol Lett* .2017;39(2) : 10.1007/s10529-016-2244-7 339-350 10.1007/s10529-016-2244-7 27812821PMC5247545

[2] : Adherent but Not Suspension-Cultured Embryoid Bodies Develop into Laminated Retinal Organoids. *J Dev Biol* .2021;9(3) : 10.3390/jdb9030038 10.3390/jdb9030038 34564087PMC8482155

[3] : 3D culture of human pluripotent stem cells in RGD-alginate hydrogel improves retinal tissue development. *Acta Biomater* .49: 10.1016/j.actbio.2016.11.016 329-343 10.1016/j.actbio.2016.11.016 27826002

# Peer review: Reviewer response for version 1

**DOI:** 10.5256/f1000research.120258.r128030

**Published:** 2022-04-21

**Authors:** Magdalene J. Seiler

**Affiliations:** 1 Stem Cell Research Center and Department of Physical Medicine & Rehabilitation and Department of Anatomy & Neurobiology and Department of Ophthalmology, Gavin Herbert Eye Institute, University of California, Irvine, CA, USA

This paper aimed to develop an improved 3D retinal organoid differentiation method from human induced pluripotent stem cells (iPSCs). Goal was to reduce the variability of the developing organoids, concentrating on early stages of development (up to d50). The main improvement was to obtain a uniform size of embryoid bodies (EBs) by using forced aggregation of iPSCs in aggrewells. EBs were then maintained in suspension culture. The neural induction media up to d18 were defined containing KOSR (knock-out serum residue). However, 10% FBS (fetal bovine serum) was used for the retinal differentiation media from d18.

 The authors determined that a smaller EB size (160µm) was optimal for differentiation of optic vesicles. However, it was impossible to control the size of the optic vesicles. Organoids were analyzed at d20, 35 and 50 by qPCR and immunohistochemistry (no qPCR data for d50).

 In general, the paper is well written, with good figures. The documentation is excellent. The reviewer has only few comments:

1. Three iPSC cell lines derived from 3 male volunteers were used – Why only male cell lines? The rationale for this should be explained.
2. Another question: Aggrewells and other methods to obtain EBs of a specific size have been used by many different researchers (only some of them cited in the paper). Thus, the method used is not that new.
3. Why no qPCR data for d50?

Is the rationale for developing the new method (or application) clearly explained?

Yes

Are the 3Rs implications of the work described accurately?

Yes

Is the description of the method technically sound?

Yes

If applicable, is the statistical analysis and its interpretation appropriate?

Yes

Are the conclusions about the method and its performance adequately supported by the findings presented in the article?

Yes

If any results are presented, are all the source data underlying the results available to ensure full reproducibility?

Yes

Are a suitable application and appropriate end-users identified?

Yes

Are sufficient details provided to allow replication of the method development and its use by others?

Yes

Reviewer Expertise:

Retinal organoids, retinal development, retinal degeneration, retinal transplantation

I confirm that I have read this submission and believe that I have an appropriate level of expertise to confirm that it is of an acceptable scientific standard.
